# Comparison of PET/CT and whole-mount histopathology sections of the human prostate: a new strategy for voxel-wise evaluation

**DOI:** 10.1186/s40658-017-0188-1

**Published:** 2017-08-17

**Authors:** F. Schiller, T. Fechter, C. Zamboglou, A. Chirindel, N. Salman, C.A. Jilg, V. Drendel, M. Werner, P.T. Meyer, A.-L. Grosu, M. Mix

**Affiliations:** 1 Department of Nuclear Medicine, University Medical Center Freiburg, Hugstetter Str. 55, 79106 Freiburg, Germany; 2 Department of Radiation Oncology, University Medical Center Freiburg, Robert-Koch-Str. 3, 79106 Freiburg, Germany; 3 Department of Nuclear Medicine, Claraspital Basel, Kleinriehenstr. 30, 4058 Basel, Switzerland; 4 Department of Urology, University Medical Center Freiburg, Hugstetter Str. 55, 79106 Freiburg, Germany; 5 Department of Pathology, University Medical Center Freiburg, Breisacher Str. 115A, 79106 Freiburg, Germany; 6 German Cancer Consortium (DKTK) Partner Site, Freiburg, Germany

**Keywords:** Prostate cancer, PSMA PET/CT, Histopathology, Voxel-wise, Coregistration

## Abstract

**Background:**

Implementation of PET/CT in diagnosis of primary prostate cancer (PCa) requires a profound knowledge about the tracer, preferably from a quantitative evaluation. Direct visual comparison of PET/CT slices to whole prostate sections is hampered by considerable uncertainties from imperfect coregistration and fundamentally different image modalities. In the current study, we present a novel method for advanced voxel-wise comparison of histopathology from excised prostates to pre-surgical PET. Resected prostates from eight patients who underwent PSMA-PET/CT were scanned (ex vivo CT) and thoroughly pathologically prepared. In vivo and ex vivo CT including histopathology were coregistered with three different methods (manual, semi−/automatic). Spatial overlap after CT-based registration was evaluated with dice similarity (DSC). Furthermore, we constructed 3D cancer distribution models from histopathologic information in various slices. Subsequent smoothing reflected the intrinsically limited spatial resolution of PSMA-PET. The resulting histoPET models were used for quantitative analysis of spatial histopathology-PET pattern agreement focusing on *p* values and coefficients of determination (*R* ^2^). We examined additional rigid mutual information (MI) coregistration directly based on PSMA-PET and histoPET.

**Results:**

Mean DSC for the three different methods (ManReg, ScalFactReg, and DefReg) were 0.79 ± 0.06, 0.82 ± 0.04, and 0.90 ± 0.02, respectively, while quantification of PET-histopathology pattern agreement after CT-based registration revealed *R* ^2^ 45.7, 43.2, and 41.3% on average with *p* < 10^−5^. Subsequent PET-based MI coregistration yielded *R* ^2^ 61.3, 55.9, and 55.6%, respectively, while implying anatomically plausible transformations.

**Conclusions:**

Creating 3D histoPET models based on thorough histopathological preparation allowed sophisticated quantitative analyses showing highly significant correlations between histopathology and (PSMA-)PET. We recommend manual CT-based coregistration followed by a PET-based MI algorithm to overcome limitations of purely CT-based coregistrations for meaningful voxel-wise comparisons between PET and histopathology.

## Background

Molecular imaging (positron emission tomography, PET) can detect primary PCa with high sensitivity/specificity [1, 2] and can provide an objective tool for target delineation by dose-painting [3]. For introduction of a new PET tracer in diagnosis and treatment planning of primary PCa, profound knowledge about tracer accumulation in PCa and non-PCa tissue is necessary. This can be achieved only by voxel-wise examination of the tracer’s performance within the prostate.

Comparisons of prostate histopathology and PET/CT data are a challenging issue. Due to complex deformations that can occur during prostatectomy and histopathologic work-up, the prostate may undergo non-linear deformation [4]. Additional uncertainties occur by slice-cutting, which often cannot precisely follow the axial plane of the in vivo PET/CT scan. Furthermore, a difference in the resolution of the available information between PET/CT imaging (resolution and slice thickness in millimeters) and histopathology (planar resolution in microns but highly incomplete axial sampling) induces further challenges in terms of coregistration [4] as well as of interpretation.

Several groups have compared the spatial distribution of a PET tracer with histopathology after prostatectomy [5–8]. In these previous trials, direct visual coregistration between PET/CT data and pathologic slices was performed. Interpretation of tracer distribution was done visually, describing sensitivity and specificity for each individual patient/lesion. Some groups divided the prostate into several sectors to compare the PET/CT findings with histopathology [8, 9]. Bundschuh et al. introduced an intermediate step in the coregistration of histopathology and in vivo PET by introducing an ex vivo CT of the resected prostate. The tumor volume was delineated in whole-mount prostate slices manually. Then, the delineated tumor volume was enlarged by applying the individual scaling factor, and the PET images were visually compared with the histopathologic findings [10]. Recently, one study performed ex vivo CT of the resected prostate in a localizer. Using the grids of the localizer as markers, pathology sections were cut at the same angle as the ex vivo CT slices. Consequently, the pathologic slices and the ex vivo CT slices corresponded. The spatial overlap between PET pattern (defined as manually delineated tumor volume) and histopathology was measured [11].

In our study, we present a new approach to voxel-wise comparison of PET/CT images with histopathologic sections. Starting after prostatectomy with a procedure similar to that described by Grosu et al. [11] to match the histopathologic specimen with ex vivo CTs, we introduce modeling of a 3D histopathology dataset taking into account the physical properties of PET (histoPET). A two-staged coregistration protocol was implemented: CT-based coregistration between in vivo CT and ex vivo CT scans followed by rigid mutual information (MI) coregistration between histoPET and in vivo PET. We determined the pattern agreement between histoPET and in vivo PET using coefficients of determination (*R* ^2^) as a new voxel-based method for quantitative comparison of histopathologic data and PET signal.

## Methods

Eight patients with histopathologically proven primary PCa (biopsy) received pre-therapeutic PSMA-PET/CT followed by radical prostatectomy (Table 1). The retrospective analysis was approved by the local ethics committee.

### PET/CT

In vivo PET/CT scans were acquired either on a 64-channel GEMINI TF PET/CT or on a 16-channel GEMINI TF BIG BORE PET/CT (both Philips Healthcare, Cleveland, OH, PET pixel size x,y,z: 2 × 2 × 2 mm) which provide virtually identical image characteristics [12]. To ensure comparability of the measurements, the two scanners were cross-calibrated. At the time of the PET scan, a contrast-enhanced diagnostic CT (120 kVp, 100–400 mAs, dose modulation, pixel size x,y,z: 1.172 × 1.172 × 2 mm) was performed.

### Ex vivo imaging and histopathology

**Fig. 1:**
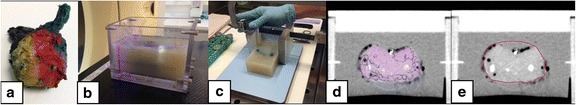
Histopathological preparation and ex vivo CT scans. **a** Resected prostate marked with special ink for later orientation. **b** Prostate embedded in agarose inside a special localizer box. **c** Cutting at defined marker positions. **d** Overlay of ex vivo CT with histopathological information (*pink*) including tumor definition (*black* lines). **e** Ex vivo CT with contours for coregistration

After open radical retropubic prostatectomy and 24-h formalin fixation, the basic edges (ventral, dorsal, left, and right) of the resected prostate (see Fig. 1a) were marked with special ink to support orientation of the prostate in the agarose-filled cuvette and in the XYZ space of ex vivo CT. Radiopaque plastic pipes were inserted into the prostate for additional visual control between histopathologic slices and CT. The resected prostates were embedded in 6.5% agarose in a localizer with a 4-mm marker profile (see Fig. 1b).

The ex vivo CT scan was performed by means of planning CT for radiation oncology treatment (16-channel Phillips Brilliance Big Bore) using 120 kV and 100 mAs (pixel size x,y,z: 0,3 × 0,3 × 2 mm).

Every 4 mm, 2-μm-thick slices were cut at the same angle and position as the CT slices using a cutting device (Fig. 1c). Subsequently, the remaining pipes were removed from the slices, and a visible cavity remained which was still visible after histopathologic preparation. Pathologic work-up involves staining of the PCa with hematoxylin and eosin and delineation of PCa on every slice using black ink (Fig. 1d). Delineation was performed by an experienced pathologist manually supported by morphological patterns of healthy and malignant prostate tissue. Each slice was scanned with a CanoScan 9000F MarkII (Canon).

The histopathological information (see Fig. 1d, pink overlay) including tumor definition (black lines) was matched to the ex vivo CT scan. The contour of the prostate, the overlap between radiopaque pipes in CT and pipe cavities in histopathologic slices, and the markers at the localizer wall served as guidance for coregistration. VOIs were delineated within the ex vivo CT representing the PCa as well as the prostate where complete contours were estimated in case of resected prostate parts (Fig. 1e, red line).

**Table 1:** Datasets

	Age (years)	PSA (ng/ml)	TNM	Gleason score	PCa (% of prostatic tissue)
1	67	6.07	pT3a pN1 cM0	3 + 4 (7a)	28
2	52	51.13	pT3b pN1 cM0	5 + 4 (9)	42
3	59	9.15	pT2c pN0 cM0	4 + 3 (7b)	4
4	60	49	pT2c pN1 cM0	3 + 4 (7a)	56
5	49	5.57	pT2c pN0 cM0	3 + 3 (6)	4
6	62	47.17	pT3b pN1 cM0	4 + 4 (8)	62
7	74	8.82	pT2c pN0 cM0	3 + 4 (7a)	3
8	61	10.57	pT2c pN0 cM0	3 + 4 (7a)	15
Mean	60.50	23.44			27
SD±	7.87	21.34			24.12

The portion of malignant tissue in the prostate was determined after prostatectomy, based on the areas of the malignant and total prostate tissue in the histopathologic slices (Fig. 1)

### Coregistration

The following main steps were performed:

I. Coregistration of histopathology and ex vivo CT including PCa delineation
II. Coregistration between in vivo and ex vivo CT scans
III. Modeling of 3D histoPET images based on the coregistered histopathology
IV. Coregistration procedures including PET information

I. Coregistration of histopathology and ex vivo CT including PCa delineation

In a manner similar to the procedure described by Grosu et al. [11], whole-mount prostate slices were coregistered to the ex vivo CT. In the current work, we used an improved fixation device (localizer) consisting of a customized cuvette with 4-mm-spaced markers, filled with agarose in which the prostate was embedded and fixated. After ex vivo CT scan of the localizer, the pathologic slices were cut perpendicular to the urethra and along the localizer markers using a customized cutting device. Thus, the sections obtained had the same cutting angle as the corresponding ex vivo CT slices (for detailed explanation see Fig. 1). Subsequently, ex vivo CT was displayed using the Medical Imaging Interaction Toolkit (MITK, [13]) and the entire prostatic gland was contoured. Matching between histology slices and ex vivo CT images was done visually in MITK. Once the coregistration of histology slices to ex vivo CT images was performed, the pathologic contours were transferred onto the CT images, and expanded by 2 mm in both Z axis directions to cover the volume in between the 4-mm-spaced histological cuts.

II. Coregistration between in vivo and ex vivo CT scans

Three methods of CT-based coregistration were compared:

1. Manual coregistration (ManReg)
  Ex-vivo CT was manually (ManReg) coregistered to in vivo CT, using MITK software, based on a consensus of two independent observers. In the first step, the prostatic gland was delineated in the in vivo CT by using soft-tissue windowing (window level: 40–70 HU, window width: 100–200 HU). Ex vivo CT was oriented in the XYZ space of the in vivo CT by using the marker profile of the localizer. The axes between the apex and the prostatic base in ex−/ in vivo CT guided further coregistration, and rotation was used for final alignment. The delineated contours of the prostatic glands in ex vivo and in vivo CT served as reference points for anisotropic scaling of the ex vivo prostate, which was performed manually in all three dimensions. The transformations/deformations of the coregistration steps were also applied to the VOIs defined on the ex vivo CT in step I.
2. Manual coregistration with automatic scaling factor (ScalFactReg)
  Manual coregistration as described in method 1, but with isotropic scaling of the ex vivo CT using a derived scaling factor to compensate for the prostate shrinking after prostatectomy. This scaling factor was calculated based on in vivo and ex vivo prostate volumes by:
$$ \mathrm{scalingFactor}=\sqrt[3]{V_{\mathrm{vivo}}/{V}_{\mathrm{vitro}}} $$,
  where *V*_vivo_ is the volume of the whole prostate gland on in vivo CT and *V*_vitro_ is the volume of the delineation on the ex vivo CT.
3. Deformable coregistration (DefReg)
  To cover non-affine deformations after prostatectomy, in vivo and ex vivo CT were coregistered by a deformable coregistration algorithm. As no internal structure of the prostate is visible on CT and the prominent drainage pipes on the ex vivo CT may lead to false correspondences an outline-based algorithm was chosen [14]. The algorithm simultaneously calculated correspondences and non-affine transformations between the outline points. Point correspondences were determined by so-called softassign, and the deformations by thin-plate spline method. As starting point, the delineations of in vivo and ex vivo CT were used and parameters were set to cover all possible point correspondences.
  For the assessment of the performance and determination of spatial overlaps of CT-based coregistration approaches, the Dice Similarity Coefficient (DSC) was used: DSC = 2 ∨ *A* ∩ *B* ∨ (|*A*| + |*B*|). *A* represents the prostate contour done on the in vivo CT and *B* the prostate contour done on the ex vivo CT after coregistration.

III. Modeling of 3D histoPET images based on the coregistered histopathology

According to the PCa distribution established in step I, a value of 1 was assigned to every voxel classified as tumor volume, which may be interpreted as a histopathology-based tumor likelihood of 1. As the tracer PSMA binds to healthy prostate tissue as well, although to a much lesser extent, non-tumor voxels were set to 0.1. We estimated that the intra-tumor variability of PSMA accumulation is low in relation to the difference between healthy and malignant tissue, justifying a ‘binarized’ model within the prostate volume. Remaining voxels outside the prostate were assigned a value of 0.

**Fig. 2:**
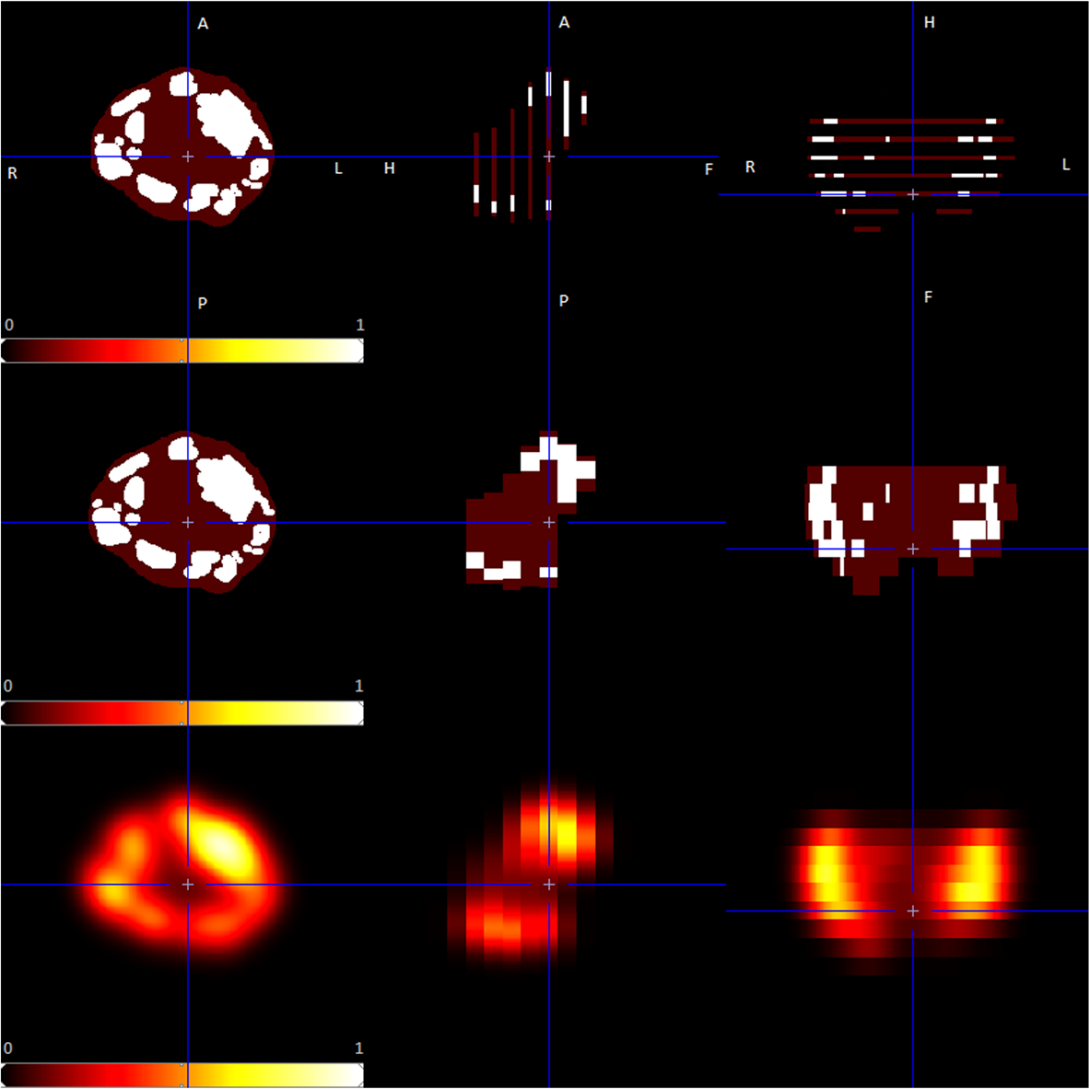
Procedure for constructing a simulated PET image (histoPET). The histoPET was created from the histopathologic information matched to ex vivo CT (axial, sagittal, and coronal views from left to right). Delineation of PCa (=“1”) and non-neoplastic prostate tissue (=“0.1”) was performed at every histopathologic slice (*top row*). Note that the transaxial slice thickness of the pathological slices in this row was increased to 1 mm (from originally 2 μm) for illustration purposes only. The discretized histopathological slices were then used to approximate the entire PCa volume in a 4-mm slice (distance of histopathologic slices, middle row, see also step I) and smoothed in order to account for the limited spatial resolution of PET scans (*bottom row*, see also step III)

To take into account the limited spatial resolution of PET (including the positron range of ^68^Ga, [15]) compared to histology, a Gaussian smoothing of the histological 3D information with an FWHM of 7 mm using the PMOD software package (version 3.6, PMOD Technologies Ltd.) was performed. This led to the so-called ‘histoPET’, corresponding to a modeled PET image implied by the given histopathologic tumor distribution (Fig. 2). The unit of these histoPET values was called ‘relSUV’ (in analogy to SUV in PET), which may be interpreted as smoothed tumor likelihood.

IV. Coregistration procedures including PET information

We added rigid mutual information (MI) coregistration of the in vivo PET scan and the modeled histoPET to our workflow. Each of the three CT-based coregistration procedures was used as starting point for matching histoPET and PSMA-PET/CT by MI where the results are named ManRegMI, ScalRegMI, and DefRegMI accordingly. MI was done with the “normalized mutual information” algorithm in PMOD v3.6 applying rigid transformations. For this purpose, masks were applied to the PET in order to take into account PET information only from the prostate. These masks were defined using anatomical CT information while sparing regions affected by high tracer accumulation in the bladder as visible in the PET image.

PET coregistration needed to be done carefully, since a reasonable agreement for simpler patterns can easily be found if the PETs are just shifted or rotated far enough. We estimated that shifts of up to two FWHMs of the PET resolution (i.e. 14 mm) can be considered still plausible. Measurement of transformations (shift/rotation) after MI coregistration was performed in PMOD. Furthermore, visual evaluation ensured that MI resulted in anatomical plausible transformations. The alignment of in vivo CT and PET scan was already given by the hardware coregistration of the combined PET/CT scanners.

### Voxel-wise analysis

The spatial overlap between patterns of histoPET and PSMA-PET before and after MI was compared visually and quantitatively. PET signals from prostate regions which could not be examined histopathologically would bias the results. Thus, the statistical analysis needed to be derived from VOIs excluding such regions (similar to the histoPET-PET MI coregistration procedure, step IV in the coregistration workflow). Subsequently, SUV (PSMA-PET) and relSUV (histoPET) values for each voxel within the VOI were measured and linear regressions yielded coefficients of determination (*R* ^2^) as well as *p* values (*t* statistics, MATLAB R2014a) which were visualized by scatter plots.

## Results

### Assessment of CT-based registration

For all patients the average DSCs for ManReg, ScalFactReg, and DefReg were 0.79 ± 0.06, 0.82 ± 0.04, and 0.90 ± 0.02, respectively. Wilcoxon matched-pairs signed-rank test showed no significant difference between ManReg and ScalFactReg (*p* = 0.219), whereas DSCs were significant higher after DefReg compared to ManReg (*p* = 0.008) and ScalFactReg (*p* = 0.008), respectively.

### Plausibility test of MI coregistration

**Fig. 3:**
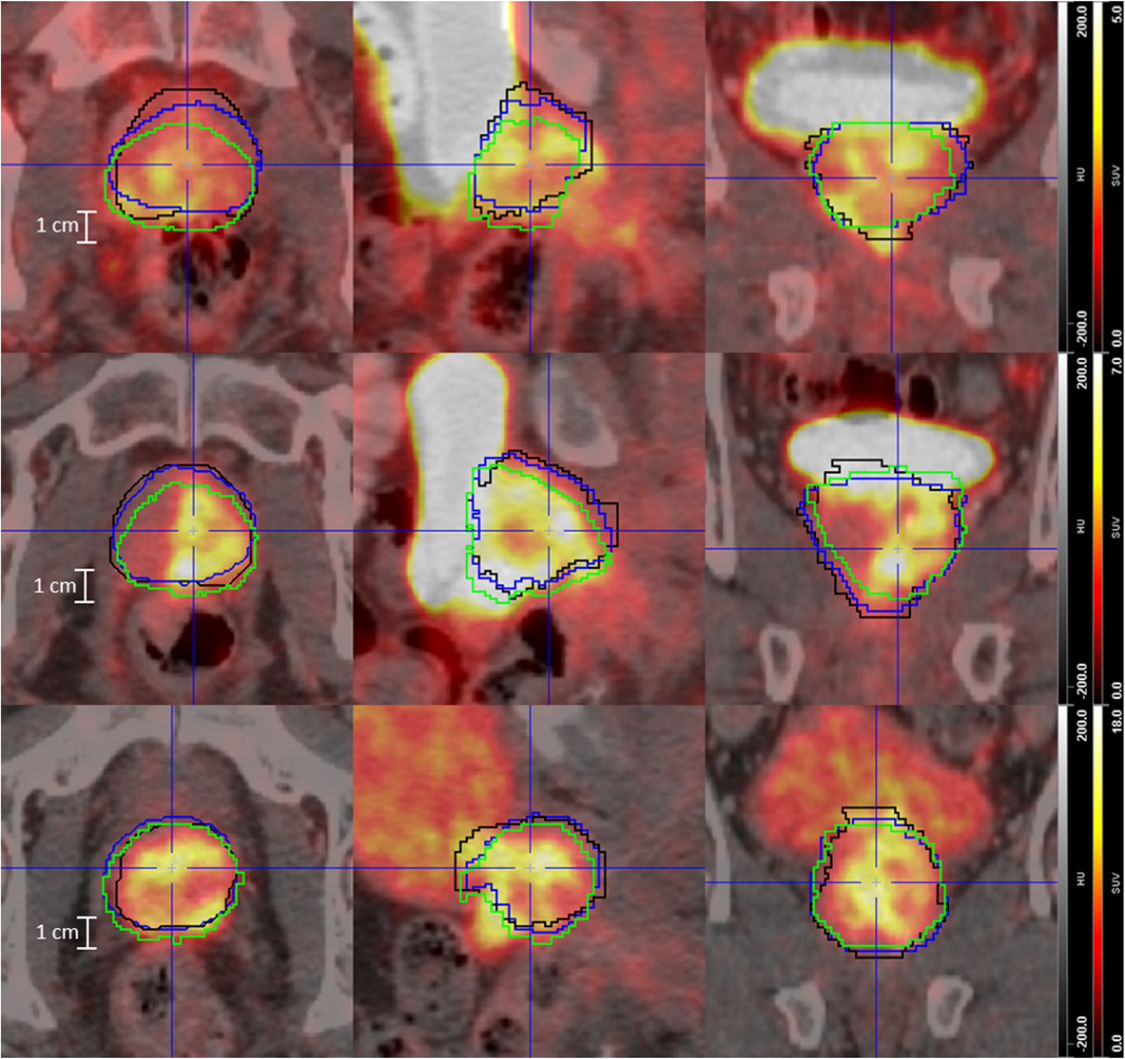
Localization of the prostate according to ManReg and ManRegMI. PSMA-PET/CTs for patient 1 (*top*), 2 (*middle*), and 3 (*bottom*) in axial, sagittal, and coronal views each (from left to right). The images show the CT-based delineation of the in vivo prostate (*black*), the coregistered ex vivo contour (*blue*, ManReg method), and the result of applying the PET-based MI transformation onto the latter (*green*, ManRegMI). The largest shift (about 7 mm) in case of patient 1 places the ex vivo contour slightly into the rectum according to the CT image. However, the MI algorithm still yields a well-justified transformation, as such a shift can be easily explained as an inevitable consequence of a misalignment between CT and PSMA-PET (see Discussion)

The 3D shifts and rotations implied by MI ranged from 2 to 10 mm and from 0 to 8° in all cases but one (Table 2). One transformation based on the DefReg coregistration of patient 8 implied a high shift/rotation of 18 mm/14° due to a pronounced deformation of the original DefReg contour. Visual assessment revealed anatomically plausible transformations with MI (Fig. 3).

**Table 2:** Figures of merit for CT-based and subsequent MI coregistration

Patient	CT-based coregistration			Subsequent MI		
	Method	DSC	*R* ^2^ [%]	*R* ^2^	Parameters (3D)	
					Shift [mm]	Rotation [°]
1	ManReg	0.78	20.1	45.8	7	0
	ScalFactReg	0.78	12.4	37.5	8	0
	DefReg	0.91	21.9	42.4	7	0
2	ManReg	0.86	40.1	54.5	7	8
	ScalFactReg	0.86	41.2	54.1	7	0
	DefReg	0.91	34.3	48.3	9	1
3	ManReg	0.83	64.5	80.7	3	2
	ScalFactReg	0.85	67.3	74.6	3	2
	DefReg	0.92	50.8	62.3	6	1
4	ManReg	0.72	49.3	62.1	6	1
	ScalFactReg	0.75	63.4	62.6	6	1
	DefReg	0.86	57.0	57.9	6	1
5	ManReg	0.73	45.2	56.4	6	0
	ScalFactReg	0.85	37.7	41.4	10	2
	DefReg	0.91	49.2	52.7	9	0
6	ManReg	0.87	66.6	80.4	4	4
	ScalFactReg	0.83	58.9	78.2	4	1
	DefReg	0.92	67.9	79.1	3	3
7	ManReg	0.73	29.0	42.5	8	0
	ScalFactReg	0.81	26.5	35.5	8	0
	DefReg	0.89	11.4	40.0	8	0
8	ManReg	0.83	51.1	68.2	2	10
	ScalFactReg	0.84	37.8	63.4	4	0
	DefReg	0.91	37.8	62.5	18	14
Mean	ManReg		45.7	61.3		
	ScalFactReg		43.2	55.9		
	DefReg		41.3	55.6		

DSC and *R* ^2^ for CT-based coregistration methods (left part of the table). On the right-hand side, the *R* ^2^ value according to subsequent PET-based MI coregistration is shown

### Voxel-wise analyses

**Fig. 4:**
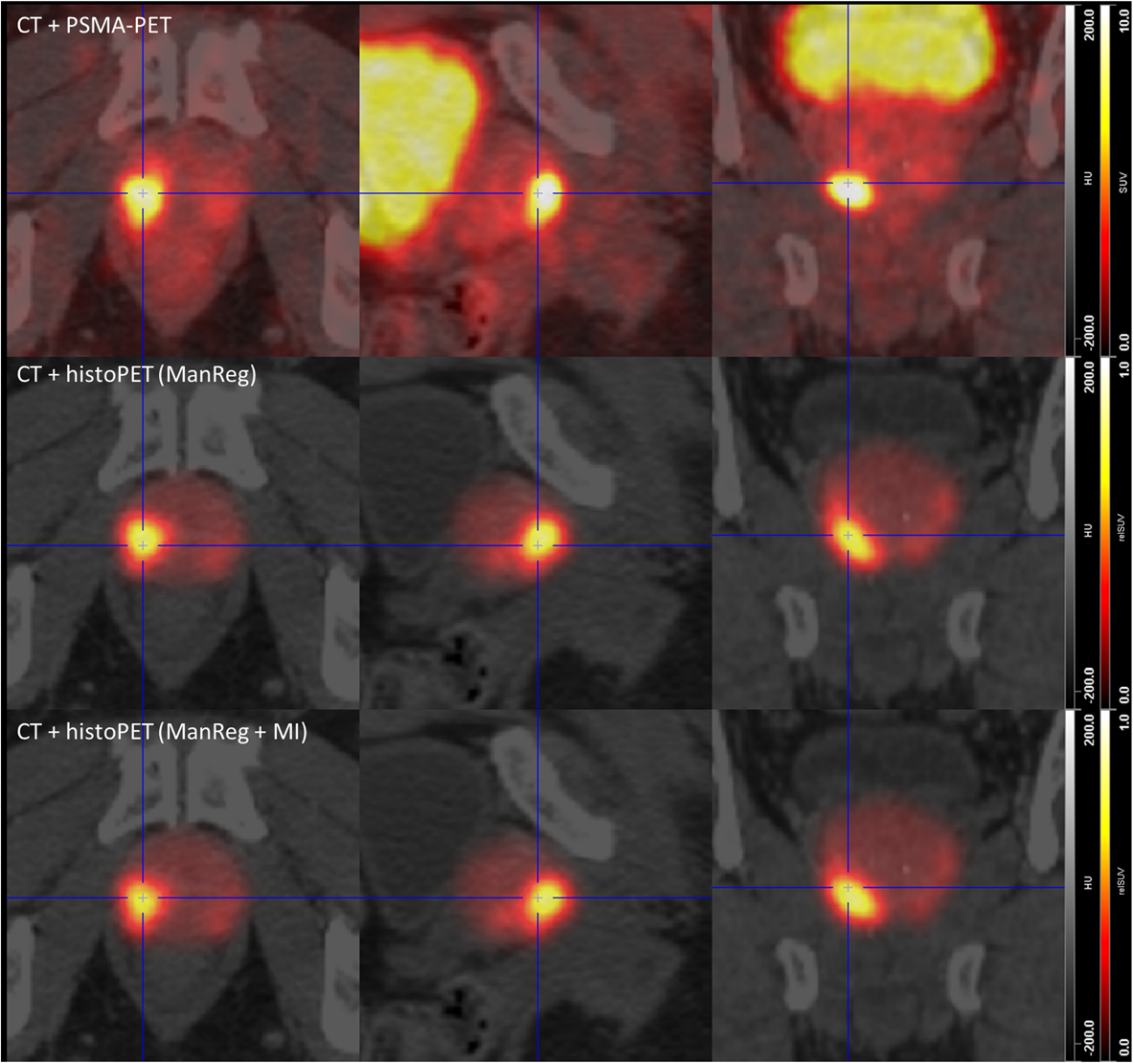
Visual comparison of PSMA-PET/CT and histoPET models according to ManReg and ManRegMI coregistration. The ManReg coregistration already shows a very good agreement between PSMA-PET and histoPET (*R* ^2^ = 64.5%). Further PET-based MI coregistration results mainly in a slight shift of the histoPET in the dorsal direction (comparing rows 2 and 3) and provides a considerably better agreement (*R* ^2^ = 80.7%). For better orientation, the blue crosshair is always located at the same anatomical position

**Fig. 5:**
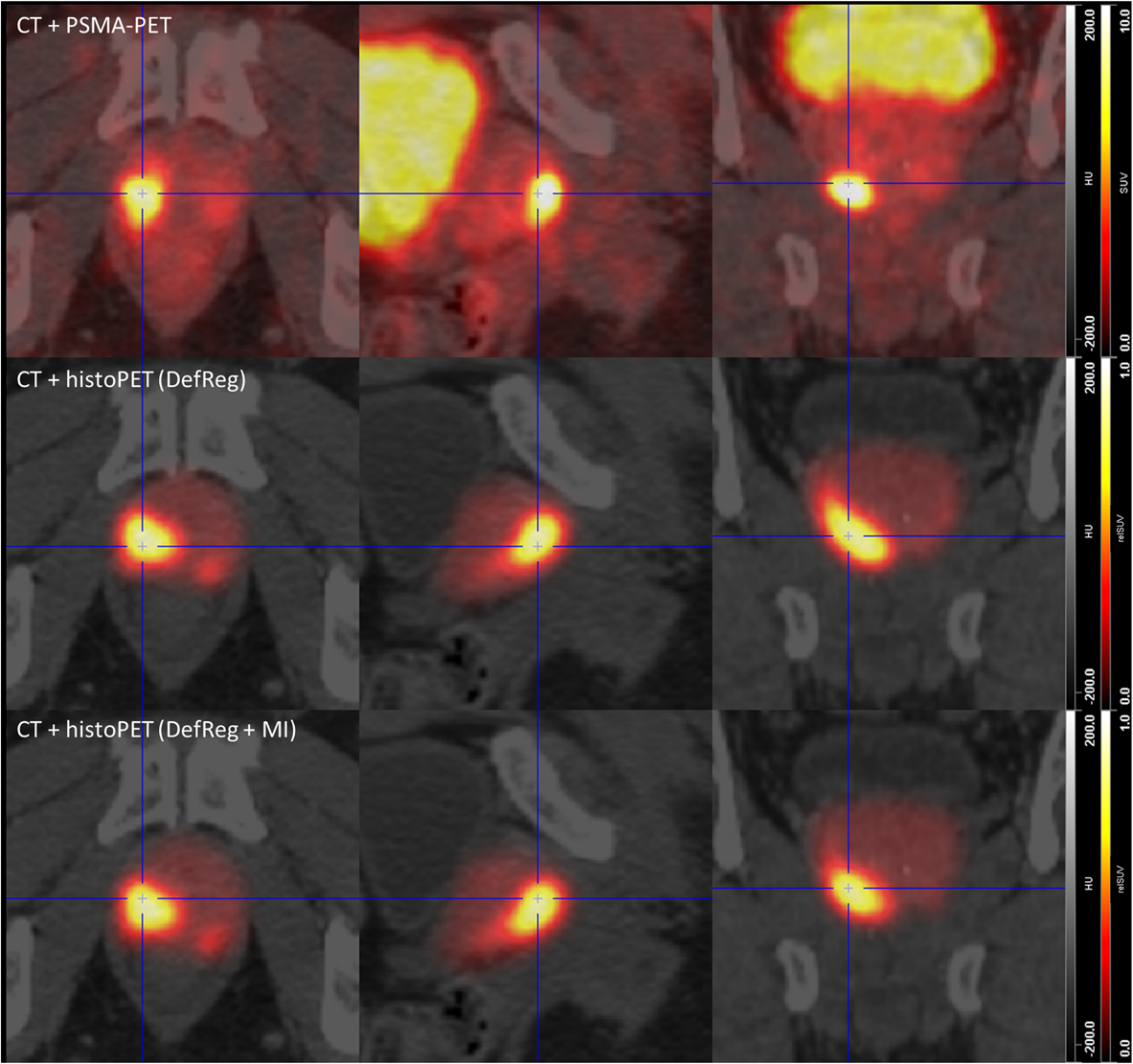
Visual comparison of PSMA-PET/CT and histoPET models according to DefReg and DefRegMI coregistration. The deformation of the 3D histoPET by the deformable registration algorithm is clearly visible. Results showed that DefReg is more sensitive to uncertainties in contours of the prostate on in vivo and ex vivo CT than the pure manual ManReg is. However, pattern agreement between PSMA-PET and histoPET with *R* ^2^ values from 50.8 to 62.2% without and with MI coregistration step were achieved

After CT-based coregistration, we were able to determine coefficients of determination (*R* ^2^) between PSMA-PET (SUV) and histoPET (relSUV) information which were in the range from 20.1 to 66.6%, 12.4 to 67.3%, and 11.4 to 67.9% for ManReg, ScalReg, and DefReg, respectively. Furthermore, highly significant correlations between PET and histoPET were found in all eight patients, with *p* values equal to zero (acc. to MATLAB). After MI, we found *R* ^2^ values between PSMA-PET (SUV) and histoPET (relSUV) 42.5–80.7%, 35.5–78.2%, and 40.0–79.1% for ManRegMI, ScalRegMI, and DefRegMI, respectively. A detailed visual comparison of CT, PET, and histoPET for the ManReg(MI) and DefReg(MI) coregistration for patient 3 is given in Figs. 4 and 5, respectively.

**Fig. 6:**
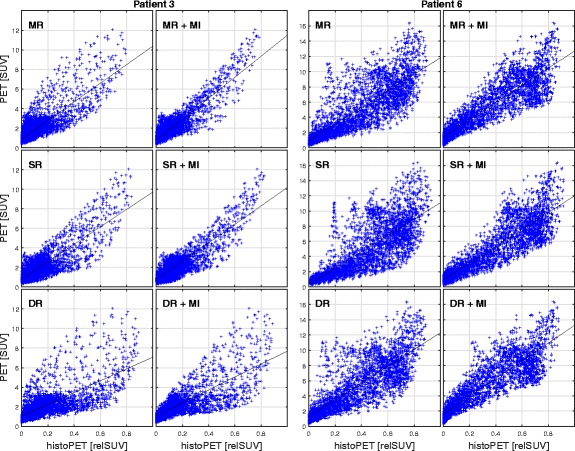
Detailed pattern comparison of PSMA-PET and histoPET—example. The *scatterplots* present the correlations of voxels within the prostate region for patients 3 (*left* columns) and 6 (*right* columns) covering all examined coregistration procedures ManReg (MR), ScalReg (SR), and DefReg (DR) before and after MI, respectively

**Fig. 7:**
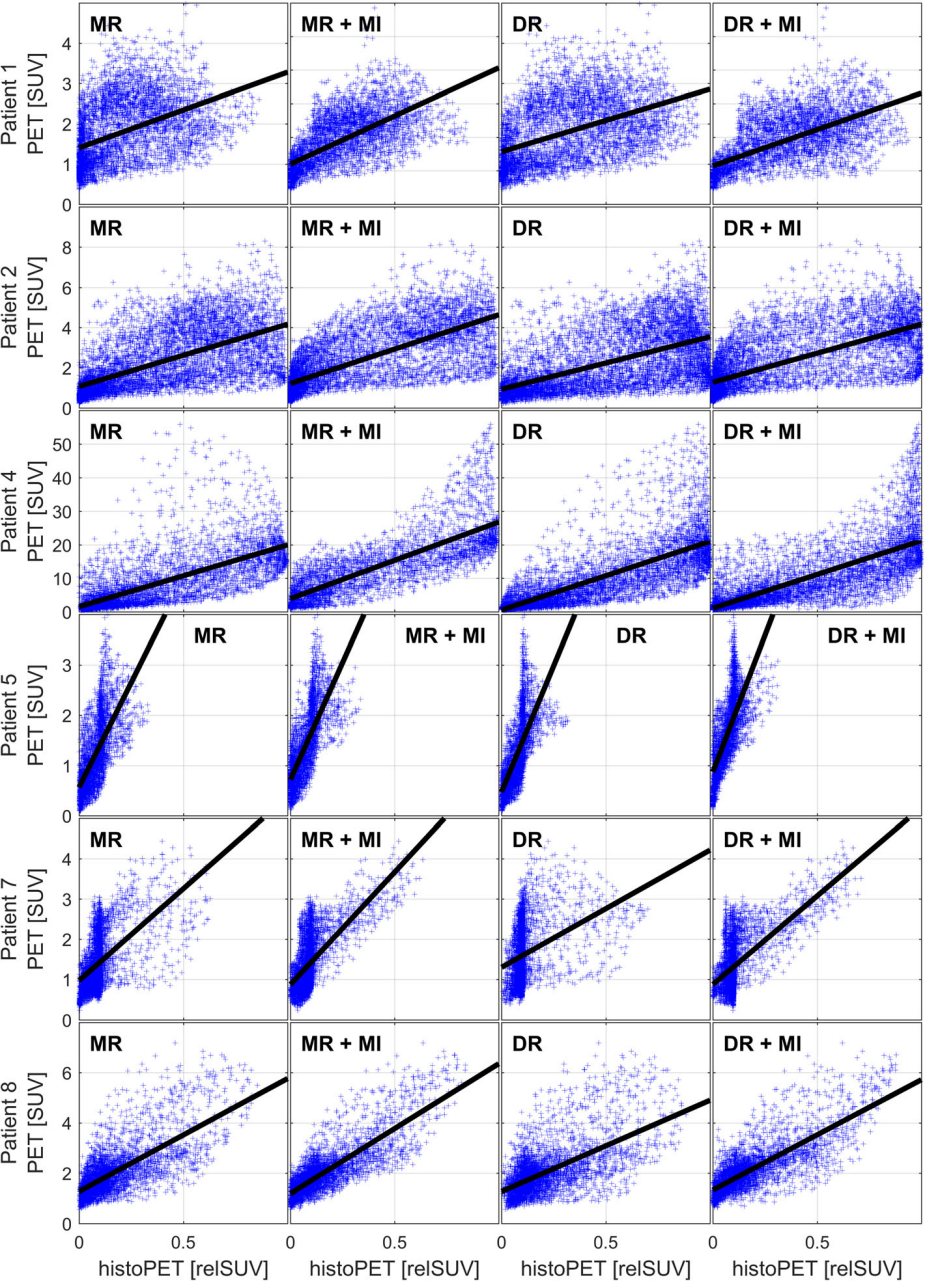
Pattern comparison—overview of remaining patients. For the six patients not presented in Fig. 6, the figure shows the correlation for the ManReg (MR, highest *R* ^2^ on average) and DefReg (DR, lowest *R* ^2^) methods before as well as after MI

Quantitative presentations are provided by Fig. 6 which covers all six coregistration procedures using the example of patients 3 and 6. The remaining six patients with their correlations after ManReg and DefReg are covered by Fig. 7.

## Discussion

The objective of this study was to present a new method for voxel-wise comparison between PET and histopathology after prostatectomy.

The commonly used procedure in evaluation of PET/CT tracers in PCa is visual comparison between whole-prostate sections and PET/CT slices [5–8]. However, one cannot compare histopathology voxel-wise with PET without accounting for the differences in resolution between PET (several mm) and histopathology (μm). This was achieved by smoothing of the created 3D histopathology model. Pattern analyses between histoPET and in vivo PET focusing on the significance (*p* value) and *R* ^2^ values of overall correlations were favored for visual comparisons of spatial PET-histopathology overlap.

Creating a PET model by smoothing of histopathologic information requires a 3D model of histopathologic information covering the entire prostate. Thus, the information from one histopathologic slice (thickness 2 μm) represents the PCa distribution over a thickness of 4 mm (distance between slices) in our model. In particular, small-sized tumors less than 4 mm in thickness could be missed in the histopathology while still generating a signal in the PSMA-PET image. Failure to account for this fact could then lead to the incorrect conclusion of the PET signal being a false positive. We assume that better histopathologic coverage of the prostate volume (technically demanding) will improve PET-histoPET agreement further. The mentioned pathologic routine preparation also impedes the definition of a histoPET model due to missing information. To some extent, this can be accounted for by neglecting pixels close (blurring effect in PET) to such missed regions in the pattern analysis.

**Fig. 8:**
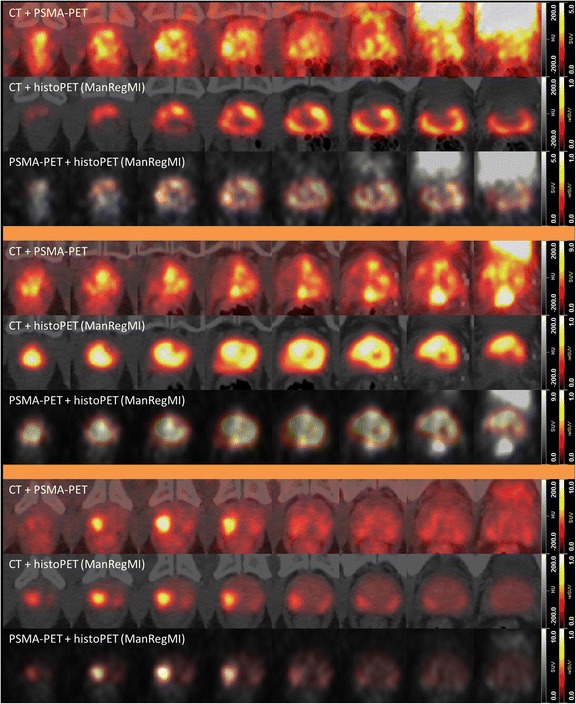
Visual agreement according to ManRegMI coregistration. Overview of axial layers (every 4 mm) throughout the prostate volume for patients 1 (*top*), 2 (*middle*), and 3 (*bottom*) for the ManRegMI coregistration. The images in each row are ordered from apex (*left*) to basis (*right*). For each patient, PSMA-PET and histoPET are presented separately from each other, with CT overlay for better orientation. The last row in each image shows the direct comparison of PSMA-PET and histoPET. Note that some discrepancies between PSMA-PET and histoPET, such as the pronounced PSMA-PET signal at the dorsal and cranial prostate region for patient 2 (*middle image*), may be due to missing histopathologic information. Such regions were excluded from the further quantitative analysis in order not to bias the results

Exclusively CT-based coregistration comprises several limitations: injuries to the prostate during surgery, shrinkage (possibly non-uniform), as well as pathologic routine preparation dissecting parts next to the bladder and seminal glands for ensuring good clinical practice. Coregistration may be complicated by an overestimation of the delineation of the in vivo prostate, as well as by missing internal structures of the prostate due to limited CT contrast regarding soft tissue. Additionally, one faces uncertainties in the hardware-based coregistration of PET and CT during the combined PET/CT examinations due to possible movement of the patient as well as bladder or rectum filling during the acquisition period of the PET scan after the CT (up to about 30 min). This suggests that anatomical/CT-CT coregistration alone is not sufficiently accurate to guarantee proper evaluation of the true PET-histopathology agreement. However, the CT-based coregistration step is a prerequisite, since it aligns in vivo with ex vivo CT, and thus histoPET, in anatomical plausible boundaries and ensures a reasonable starting point of subsequent MI coregistration. All CT-based registration methods resulted in good to excellent overlap (mean DSC 0.8–0.91) between in vivo and ex vivo information. DefReg performed best showing the highest DSC compared to ManReg and ScalFactReg, which is reasonable due to the nature of this algorithm. ManReg and ScalFactReg resulted in a similar spatial conformity. This work extends the approach of anatomical coregistration with MI coregistration, directly using the patterns of PSMA-PET and histoPET. The MI-algorithm provides a powerful means for visualization as well as for quantitative determination of the similarity of the patterns in PET and histoPET on a voxel basis. Visual inspection of PET patterns clearly reveals better agreement between PET and histoPET with the PET-based MI coregistration than with exclusive CT-based coregistration for all three tested methods. Consistently, the *R* ^2^ values considerably increase, up to 81%, implying that a large fraction of the PET variance may be explained by the histopathologic examination, despite its rather poor coverage of the prostate volume.

MI based on the purely manual CT-based coregistration method ManReg showed systematically better agreement (*R* ^2^) between PET and histoPET in all patients except patient 4 where it was similar to ScalRegMI. ScalRegMI and DefRegMI yield similar agreement with both below ManRegMI. As MI is applied after all three CT-based methods, differences in *R* ^2^ should mainly be due to different scaling/deformation of the PCa distribution. After visual assessment of the input non-MI models, we estimate that the ScalReg model may be affected by slight overestimation of the in vivo prostate volume and thus the scaling factor in combination with the applied isotropic scaling. Similarly, the algorithm controlling the DefReg coregistration seems to be extremely sensitive to the used contours where slight deviations lead to relatively pronounced deformations in the output. See Figs. 4 and 5 for the visual and Fig. 6 for a quantitative example of a corresponding situation in patient 3 in contrast to patient 6. Although less elaborate, manual or visual coregistration seems to be more stable and less susceptible to these effects (Fig. 8).

The PET-based MI coregistration needs to be performed in anatomically plausible boundaries which we estimate to shift ≤14 mm (two times the mean reconstructed image resolution of the ^68^Ga-PET scan). Still, then MI-algorithm may not find the (globally) best solution underestimating *R* ^2^. Of course, a mismatch of PET and histoPET before the MI coregistration may also be explained by a tracer accumulation inconsistent to histopathologic findings which would then mislead the MI algorithm. However, good agreement of the rather complex patterns in PET and histoPET as determined with the MI algorithm suggests that this explanation is less likely for the present cases than a mismatch due to the various uncertainties described above. In our study, only anatomically plausible transformations below or around the FWHM of PET resolution occurred (Table 1) except for DefReg(MI) in patient 8. Such transformations may be easily explained by the limitation of the hardware coregistration of PET/CT.

Our presented method enables an objective, quantitative evaluation of the spatial overlap between PET patterns and histopathology patterns, taking into account the resolution of PET. A high spatial overlap between PET and histology is necessary to justify the usage of a new PET-tracer in diagnosis and treatment planning of primary PCa. Additionally, the implemented MI coregistration step led to enhanced accuracy compared to purely CT-based coregistration. Precise coregistration between PET and histopathology enables voxel-based evaluation steps, like ROC analyses [16]. The concept of focal radiotherapy, have gained of interest for patients with primary [17] and recurrent PCa [18]. A voxel-wise examination of the tracer’s performance within the prostate is the door opener for dose-painting by numbers, which enables a heterogeneous radiation dose distribution to a voxel-level by mathematical transformations of the image information of individual voxels [3] .

## Conclusions

In this work, an advanced approach for a voxel-wise correlation between PET and histopathology was presented. Voxel-based comparisons of PET and histopathology were enabled by 3D modeling of histopathologic data adapted to PET resolution. We recommend manual coregistration between ex and in vivo CT followed by PET-based MI coregistration as a less complex method which nevertheless appears more stable and more precise compared to (semi-)automatic methods.

## Abbrevations

DefReg(MI): Deformable registration (with mutual information)
DSC: Dice similarity
HD: Hausdorff distance
ManReg(MI): Manual registration (with mutual information)
MI: Mutual information registration
PCa: Prostate cancer
*R*^2^: Coefficients of determination
ScalFact Reg(MI): Manual coregistration with automatic scaling factor (with mutual information)
